# Are Historical Buildings More Adaptive to Minimize the Risks of Airborne Transmission of Viruses and Public Health? A Study of the Hazzazi House in Jeddah (Saudi Arabia)

**DOI:** 10.3390/ijerph18073601

**Published:** 2021-03-30

**Authors:** Alaa Alaidroos, Ayad Almaimani, Ahmed Baik, Mohamed Al-Amodi, Khan Rubayet Rahaman

**Affiliations:** 1 Architectural Engineering Department, Collage of Engineering, King Abdulaziz University KAU-Rabigh, Rabigh 25732, Saudi Arabia; aalaidroos@kau.edu.sa; 2 Architecture Department, Faculty of Architecture and Planning, King Abdulaziz University (KAU), Jeddah 21589, Saudi Arabia; ealmaimani@kau.edu.sa (A.A.); masalamoudi@kau.edu.sa (M.A.-A.); 3 Geomatics Department, Faculty of Architecture and Planning, King Abdulaziz University (KAU), Jeddah 21589, Saudi Arabia; abaik@kau.edu.sa; 4 Department of Geography and Environment Studies, St. Mary’s University, Halifax, NS B3H 3C3, Canada

**Keywords:** historic buildings, airborne transmission, public health, risk management

## Abstract

The coronavirus (COVID-19) pandemic has brought immense challenges to the natural and built environment to develop an antivirus-enabled model for reducing potential risks of spreading the virus at varied scales such as buildings, neighborhoods, and cities. Spatial configurations of structures may hinder or assist the spread of viruses in the built environment. In this study, we have hypothesized that suitable air ventilation in historic buildings may enhance the built environment to combat the spreading of infectious viruses. To provide such quantitative shreds of evidence, we have generated and estimated an integrated model to summarize obtained information by considering natural ventilation, wind speed, inflow and outflow, wind direction, and forecasting the associated risks of airborne disease transmission in a historical building (i.e., the Hazzazi House in particular). Intrinsically, the results have demonstrated that the effectiveness of natural ventilation has directly influenced reducing the risks of transmitting airborne infectious viruses for the selected heritage building in Jeddah (Saudi Arabia). The adopted methods in this research may be useful to understand the potentials of conserving old heritage buildings. Consequently, the results demonstrate that natural air ventilation systems are critical to combat the spread of infectious diseases in the pandemic.

## 1. Introduction

Classifications of infection transmission modes have been reviewed in several research outcomes in the recent past. Essentially, the most reported means of transmission is found to be through human to human and airborne transmission [1,2,3]. A recent report presented by the World Health Organization (WHO) has stated that the latest novel coronavirus (COVID-19) can be transmitted through respiratory droplets and contact routes between people [4,5]. In contrast, it has been reported that SARS-COV-2 is able to survive in the air for hours during the day, which could increase the probability of airborne infection transmission [6]. As a matter of fact, SARS-COV-2 is classified as a coronavirus strain that has been developed to the novel coronavirus (COVID-19), which has been then declared as a global pandemic on 12 March 2020 [7]. In order to prevent the spread of coronaviruses, control strategies must be developed by identifying the potential impact of environmental factors, in addition to analyzing the transmission routes of these ruthless viruses. Consequently, various studies are found in the literature investigating the possibility of airborne transmission of infectious viruses, especially in confined spaces such as health care centers, quarantines, schools, and houses [8,9]. In fact, the concentration occurrence of viruses can be intensified in the air of confined spaces, thus increasing the possibility of airborne cross-infection transmission. A recent study has revealed that the SARS virus may have been transmitted in apartment buildings easily through air [10].

In contrast, ventilation systems, either natural or mechanical, are commonly recognized for introducing fresh air into the building to enhance indoor air quality. Ventilation methods in buildings are reported to play a critical role in mitigating airborne infection transmission in confined spaces. A classic study by Phair [11] has stated that ventilation systems in buildings are required to prevent hazards and eliminate airborne infectious viruses. Nonetheless, the latest studies have agreed that ventilation, in general, has a direct impact on airborne infection transmission [12,13]. A recent review of the association between human health and indoor air pollution has revealed that poor ventilation levels are observed in most residential and commercial buildings in the middle eastern countries. Moreover, this may be a major cause of the spread of airborne infections [12,13,14,15]. A comprehensive literature review presented by a multidisciplinary group of experts in medicine, public health, and engineering disciplines has summarized the association between building ventilation and transmission of airborne infection. In the scholarly review, it has revealed sufficient evidence of the relationship between ventilation in buildings and the spread of infectious diseases. However, there are a few evidences that quantify the minimum requirements of ventilation rates in buildings to prevent the spread of airborne infection transmission [16,17,18]. During the latest COVID-19 pandemic, evidences indicated that indoor environments with insufficient ventilation help transmit COVID-19 through the air, and hence supplying frequent fresh air into the enclosed space is considered an effective remedy for diluting the concentration of the virus [15]. Similarly, Carrer et al. [19] examined the available indications of the links between ventilation rates and health aspects in buildings. The available data suggest that minimum ventilation rates are necessary to avoid health complications in buildings. Additionally, the mentioned research article has stated that further studies on the relationship between ventilation and health problems in buildings are essential to develop a solid ventilation guideline for healthy indoor environments. A review paper presented by Jayaweera et al. [20] has outlined the current studies conducted on the behavior of virus-laden droplets and aerosols in several environmental settings and discussed infection possibilities in confined spaces through the virus transmission under numerous ventilation conditions. The study has stated that nosocomial infection by SARS-COV-2-laden plumes is detected in confined spaces when no adequate openings and poor ventilation is observed. On the other hand, infection risk models have been implemented along with a novel approach that estimates the viral load emitted by an infected person [14,15,20,21,22,23]. Simulation results clearly demonstrate the significant role of ventilation in controlling infections in indoor environments. Amoatey et al. [14] have investigated the impact of building ventilation systems on the transmission of SARS-COV-2 specifically implemented for the middle eastern countries. According to the findings, the authors have proposed the following solutions to minimize the transmission of SARS-COV-2: (i) improving ventilation rates in buildings, (ii) utilizing natural ventilation, and (iii) employing personalized-ventilation systems. Furthermore, the role of personalized ventilation in reducing airborne infection transmission has been explored by Xu et al. [24]. The findings suggest that personalized ventilation systems are found to be effective in preventing infection transmission in case the system is maintained at high ventilation rates to ensure the availability of fresh and clean air. Moreover, control systems and HVAC (Heating, ventilation, and air conditioning) systems configuration are known to have significant impact on the ventilation performance which have been discussed by few researchers. For instance, Morawska et al. [25] discussed the application of building controls for providing effective ventilation in order to reduce infection risk in the indoors. Besides, appropriate placement of the ventilation system’s supply and exhaust vents will guarantee sufficient dilution of contaminants and thus reducing the spread of infection [26,27,28,29,30]. While increasing ventilation rates is typically expected to minimize airborne infection risk as discussed above, there is still demand for more scientific research to understand the influence of ventilation rates on controlling the spread of viral infections [18]. A recent study also agreed that available research studies provide few evidences on how ventilation rates regimes may affect bacterial and fungal concentrations of indoor air [31].

On the other hand, extensive research work was found in the literature analyzing the relationship between airborne infection and effective ventilation strategies through numerical modeling. Some research methods focused on modeling the transport and survival of airborne micro-organisms [32], while others developed mathematical models to study the viral dynamics to explore the pathogenic features of viral infection [33]. However, according to the literature, computational fluid dynamics (CFD) was the most used analysis method to evaluate airflow patterns of several ventilation strategies and their impact on viral transmission and cross-infection in buildings, in addition to monitoring microbial spread in air [34]. In fact, CFD is considered an attractive and effective analysis method to accurately visualize airflow and air distribution in confined spaces and how contaminants and airborne infection may be transmitted through air. For instance, a recent study by Wang et al. [35] presented a numerical investigation, using CFD, of gaseous pollutant cross-transmission for single-sided natural ventilation driven by buoyancy and wind. The analysis showed that ventilation rates increase with the increase of wind speed which in turn result in lowering the risk of infection; yet in this study, the risk of infection is not quantified. Another research study conducted by Lipinski et al. [36] reviewed and evaluated the current ventilation strategies in buildings and its influence on reducing the risk of pathogen transmission using CFD. Moreover, the possibility of airborne transmission of COVID-19 was investigated through CFD simulation by providing various settings of the transport and dilution of aerosol droplets [37]. Monte-Carlo simulation was also used in this study to provide a quantitative insight to the exposure time of aerosol in public indoor environments. The performance of mechanical exhaust devices and single-sided natural ventilation has been assessed to investigate the possibility of eliminating cross-infection using CFD [38]. Moreover, Peng et al. [39] summarized a series of research studies investigating pathogen transmission based on CFD modeling and has concluded that natural ventilation techniques are the main approach to dilute pathogen concentration in air. CFD was also used to examine the distribution and the evolution of coughed droplets in an air conditioned room with two ventilation patterns [40]. Another research study presented a unique assessment of airborne contaminant transport in hospitals and laboratories by coupling CFD with multizone network software for airflow analysis [41]. Beside CFD, researchers also employed statistical models and dynamic airflow simulation analysis using EnergyPlus to analyze the impact of natural ventilation on occupant exposure to fine particle pollution in office buildings [42].

In contrast, experimental studies were conducted to investigate the risk of airborne infection in addition to validating CFD models intended to analyze ventilation systems and its relationship with viral transmission. For instance, the potential of airborne infection risk along with ventilation rates was assessed in naturally ventilated hospitals using gas tracer experimentation method [43]. Another recent research work performed both experimental and numerical analysis to analyze ventilation strategies applied for a prefabricated COVID-19 double-patient ward and its effect on airflow movement and airborne transmission [44]. Respiratory droplets were injected from two manikins in the experimental setup while droplets were modelled as particles in the CFD model. Furthermore, experiments were conducted to examine the transmission of SARS-CoV-2 through vertical plumbing piping systems of an apartment building [45]. The experiment results showed that pressure differential in airduct systems could increase the airflow rate through the ducts and assist in transmitting airborne viruses. A full-scale experimental measurement was implemented to validate a CFD model that was developed to investigate several ventilation schemes with different supply airflow rates and its impact on cleanliness recovery characteristics of indoor pollutant [46]. The authors stated that air change rate played an important role in eliminating indoor pollution.

As presented above, numerical modeling techniques such as computational fluid dynamics (CFD) are widely used to explore and assess the effectiveness of ventilation systems on eliminating the potential of airborne infection transmission in confined spaces; thus, such modeling techniques are commonly successful in visualizing airflow patterns and contaminants transport but are unable to describe how occupants are affected with the presence of these contaminants or infection spread. Accordingly, there is a lack of research on quantifying the probability of infection based on the change of ventilation rates in multi-zone buildings. Therefore, the main contribution and novelty of this study, is to present an analysis approach that is able to analyze the impact of ventilation rates on the probability of airborne infection using an existing classical mathematical model developed explicitly for predicting the probability of infection in buildings as a function of ventilation rates, number of source patients, duration of exposure and other parameters. Furthermore, ventilation rates and probability of infection are produced through hourly simulations using local weather data to explore the effect of wind speed and wind direction in addition to building orientation and window size on the ventilation rates and eventually on the probability of infection in the building. In specific, the performance of natural ventilation is evaluated for estimating the air change rates and its effect on minimizing infection risk by predicting the probability of airborne infection transmission. A historical building located in Jeddah, KSA has been selected for this study. The impact of wind speed, wind direction and windows configuration on the air change rate of the building is analyzed using a whole building energy simulation tool. Air distribution and age of air inside the building is evaluated using a commercial CFD package to approximate the duration of fresh air and air circulation performance inside the building.

### Overview of the Selected Historical Building

**Figure 1:**
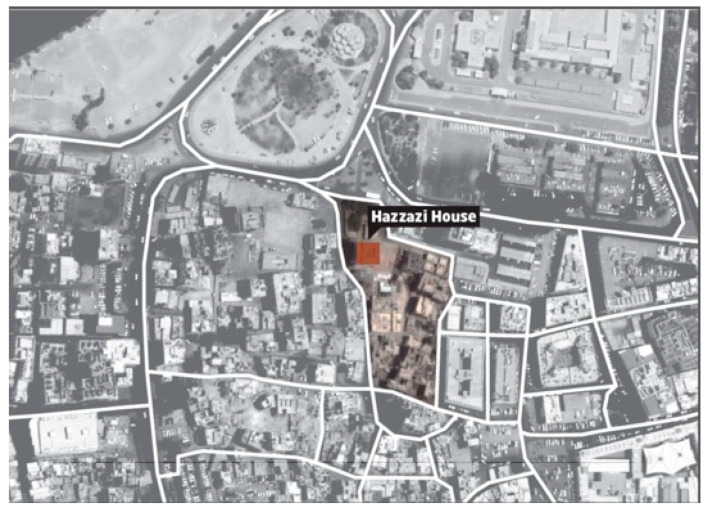
Hazzazi House Location in Old Jeddah Neighborhoods.

Saudi officials have shown an interest in assessing unoccupied heritage buildings to turn them into quarantine centers during possible health pandemics. Accordingly, due to the historical significance of the Hazzazi House and the current demand for quarantine spaces, as noted earlier, this specific building has been chosen in this research study as a model to evaluate the indoor air quality of such historical buildings. Hazazi House is one of the heritage buildings in Old Jeddah, KSA. It is located on the eastern side of Bab Jadid (the New Gate) in the Al-Sham neighborhood as shown in Figure 1. This house has stood for more than 100 years [47]. This historic building has been chosen due to its strategic location and other significant premises that are considered essential to the historical Jeddah region [47,48,49]. Additionally, Jeddah is situated on the west coast of the KSA beside the Red Sea. Its history began long before Islam, whereby the city was elevated when it was transformed into a port for Makkah City at the time of Caliph Othman bin Afan in 647AD [50,51,52,53]. Old Jeddah is composed of four parts: the Al-Sham, Al Mazloum, Al Bahr and Al Yamen neighborhoods. The urban pattern is comprised of houses, merchandise quarters, mosques and other types of buildings, penetrated by small open courts, yards and zigzagging alleyways. Hazazi House is located in historical Jeddah and has been categorized by the administration for preserving historical Jeddah as one of the first-rate buildings in terms of historical significance [48].

Hazazi House is named after the last family that lived there, who are one of the long-established families of Jeddah. They have owned the house since 1875. The house is composed of four levels, including three serviced lounges with three entrances on the ground floor, while the first, second, third and fourth floors are all comprised of private rooms, and the roof has a further three bedrooms.

Moreover, the inheritors were happy to allow the research team to investigate the house’s condition. Consequently, the building has been scanned and documented using terrestrial laser scanning (TLS) equipment thus a three-dimensional (3D) model of the historical house’s architectural and structural details has been generated. The 3D model of the Hazzazi House has then been used for further investigations and analyses as presented in this research work.

## 2. Materials and Methods

Ancient and historic buildings around the world are known for implementing natural means and passive strategies to enhance indoor thermal comfort and indoor air quality. Natural ventilation is one of these well-known techniques that has been thoroughly explored by many researchers to evaluate its effectiveness in the old passive buildings and optimizing this passive strategy to improve indoor environmental quality in new buildings.

Pressure differences is the main driving force for natural ventilation to move fresh air across the building; yet the amount and velocity of air flowing through the building depend mostly on the placement and sizes of the openings in the building [14,20,24,25,54,55]. Moreover, ventilation rate is critically dependent on the internal spaces design of the building and the size of the windows and their location. Historic residential buildings in Jeddah, for instance, are characterized by wide windows located in all perimeter and interior zones. In addition, floorplans, internal openings and narrow spaces and corridors contribute to improving the performance of natural ventilation in these buildings. While evidence shows decent ventilation rates that can be produced in these historical houses, detailed analysis is required to demonstrate the capability of natural ventilation on eliminating the risk of airborne infections. Hence, this research study is intended to evaluate the performance of natural ventilation in a selected historical residential building in Jeddah and estimate the associated ventilation rates to be able to investigate the probability of infection. The analysis of this research study is summarized as follow:

- A whole building energy model is created to simulate natural ventilation in the selected building. The effect of wind speed on average ventilation rates is presented. Cross ventilation inflow and outflow are discussed as well.
- Air change rates for several zones in different building orientations are analyzed and the impact of wind direction on the ventilation rate in the selected building is presented.
- Parametric analysis of the effect of the windows opening fraction on the ventilation rate is performed.
- Correlation between air change rates and probability of infection in the selected historical residential building is conducted and the Wells-Riley equation is used to predict the risk of airborne disease transmission according to the estimated ventilation rates of the building.
- Sensitivity analysis is implemented to examine the impact of the number of patients on the probability of infection for the naturally ventilated historical house.
- Correlations and stepwise regression analysis is performed to develop a regression model that is able to predict air change rates for the selected historical house in Jeddah using three independent variables: opening fraction of the windows, wind speed and wind direction.
- Finally, CFD analysis is conducted to investigate the airflow distribution inside the building in addition to estimating the age of air inside the building zones to guarantee the presence of fresh air.

We note that the contemporary literature has suggested that evaluation of the quanta concentration [56], and exposure risk in indoor spaces [57] are varied per capita upon considering the ventilation rate between 1.2, 3.4, 9.2, 15.7, and 20 L/s per person. However, this situation becomes evaluated in an indoor space with little or no natural ventilation and circulation systems inside the buildings. Interestingly, the heritage house we have considered here in this study has plenty of natural ventilation and air circulation system through the windows and doors. As a result, uncertainties of modelling the indoor air quality and air-borne transmission of infectious diseases remain a major challenge in an indoor environment where limited natural ventilation systems exist. In case of Hazazzi house, uncertainties of modeling indoor quality and spreading of the infectious disease may be less in compare to the exposures and aerosol transmission exist in modern buildings.

## 3. Results and Discussion

**Figure 2:**
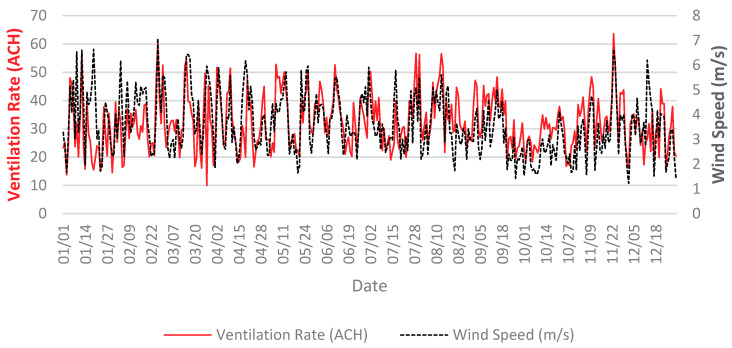
Annual average daily ventilation rate of the building and average outdoor wind speed.

The Airflow-Network model in EnergyPlus [58] was used to simulate the natural ventilation in the building. The Airflow-Network model is known for its capability of computing airflows in multizone buildings caused by wind pressure and stack effect. Several experimental studies exist in the literature were implemented to validate the model [59]. Figure 2 below shows the average daily ventilation rate (in air change per hour) against wind speed for the whole year. As expected, the average trend of ventilation rate inside the building is following the outdoor wind speed. The average ventilation rate is found to be about 20 ACH for an average annual wind speed of 4 m/s. As we can see from Figure 2, airflow rate, in general, is considered high. While the average airflow rate peaks at 64 ACH in November, the minimum airflow rate is found to be about 10 ACH in March.

**Figure 3:**
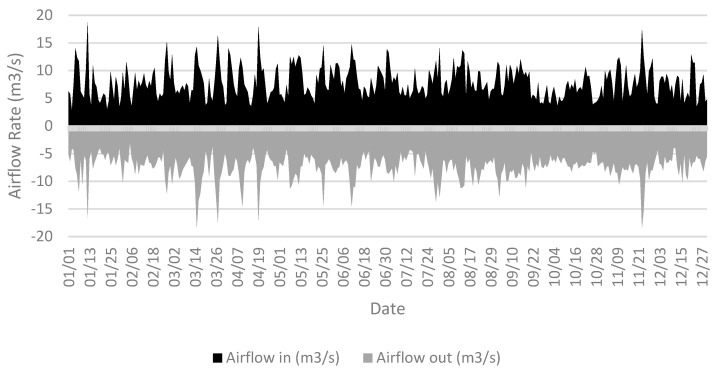
Average daily air volume flow rate out and into the building.

The large exterior window openings and the wide uniform openings between interior spaces is providing an exceptional cross ventilation conditions for the building which aid in producing high indoor air ventilation throughout the year without the assistance of mechanical means. This is depicted clearly in Figure 3, where a continuous inlet and outlet air flow rate is introduced to the building through the year which confirms ideal pressure differences around the building. In this case, the positive airflow rate values represent the airflow introduced into the building, while the negative values represent the airflow leaving the building.

**Figure 4:**
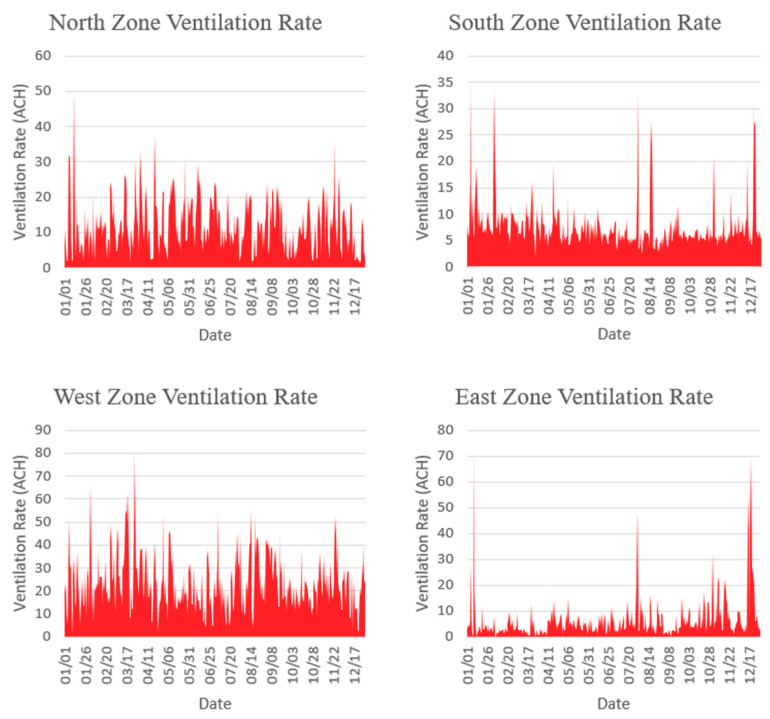
Ventilation rates for selected four zones from different orientations.

**Figure 5:**
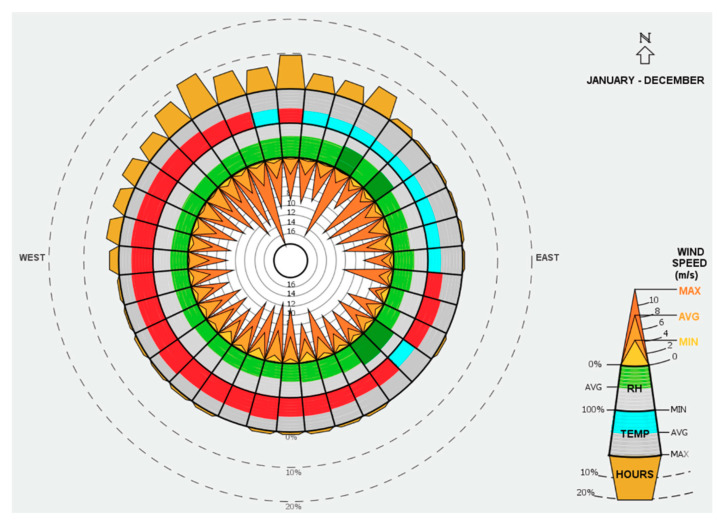
Average wind speed and wind direction in Jeddah, KSA [60].

The above ventilation rate results are considered average values of the whole building, therefore, ventilation rates for several zones located in different building orientations have been analyzed to investigate the impact of wind direction on the overall ventilation rate of the building. Figure 4 illustrates the ventilation rates of four zones from each orientation. The simulation results demonstrate high ventilation rates for the zones located in the west side of the building, while the zone located on the north sides comes second. The results presented in Figure 4 confirms the effect of wind direction on the value of ventilation rate within the building spaces. A TMY weather file is used to produce A wind wheel chart to investigate the wind direction for Jeddah. As shown in Figure 5, the majority of wind in Jeddah comes from the north west which explains the high ventilation rates on the zones located on these particular sides of the building.

Even though ventilation rates are high in the north and west orientations due to wind direction, the rest of the zones on the other sides of the building, including core zones, are receiving adequate airflow. As indicated previously, the architectural design of this historical house and many other similar houses in the historical downtown of Jeddah contribute to improving airflow distribution and ventilation rates inside the building.

**Figure 6:**
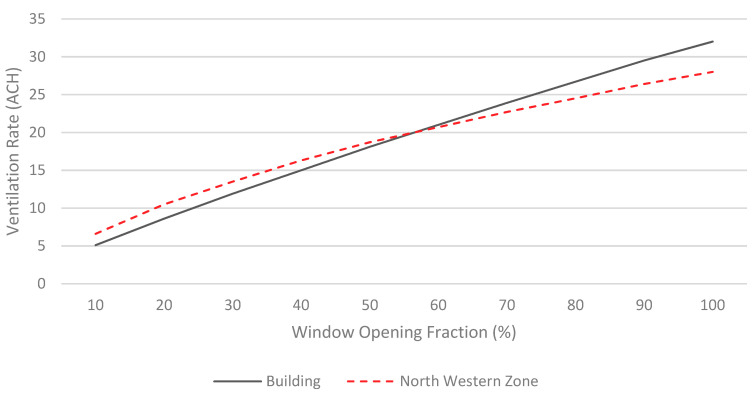
The effect of windows opening fraction on the ventilation rate of the building.

It is worth mentioning that the windows in these historical houses have no glazing, however, windows are covered with operable wooden screen covers called mashrabiyas which have been known as an ancient Arabic architectural element used to control sunlight and regulate airflow into buildings, in addition to preserving indoor privacy. Windows were open most of the time in these buildings, however, the wooden screen (mashrabiya) could impact the amount of airflow entering through the window; therefore, sensitivity analysis has been performed to evaluate the influence of the opening fraction of the windows on the ventilation rate inside the building. One zone with the highest ventilation rate located in the north western side of the building has been selected for this analysis. The window opening fraction ranges between 10% to 100% with a 10% step, where 100% opening fraction means that the window is fully open while 10% means that air can flow through 10% of the window area. As expected, ventilation rate increases with the increase of opening fraction as shown in Figure 6. Yet, the simulation results suggest a relatively high ventilation rate even with a small opening fraction such as 10% where the ventilation rate for the selected zone is 5 ACH while for the whole building is found to be about 6.5 ACH which is considered acceptable.

The ASHRAE 62.2 standard (Ventilation and Acceptable Indoor Air Quality in Residential Buildings) [61] defines the minimum air ventilation requirements for mechanical and natural ventilation systems based on the floor area of the dwelling unit. According to ASHRAE 62.2, the corresponding air ventilation rate requirement for the dwelling floor area (about 360 m^2^) is 77 L/s which is about 0.26 ACH. Apparently, the air change rate recommended by ASHRAE is way below the average air change rate in the residential building introduced in this study. As stated previously, ASHRAE 62.2 defines the general roles for minimum ventilation requirements to provide acceptable indoor air quality in residential buildings, however, these minimum requirements could not be enough to eliminate airborne infection transmission.

The World Health Organization (WHO) published a report [17] that discussed natural ventilation requirements for infection control in health-care settings. The report recommended an average ventilation rate of 160 L/s/patient for airborne precaution rooms, while 60 L/s/patient is assigned for general wards and outpatient departments. Even though the listed recommendations by WHO is offered exclusively for the health-care buildings, yet it is worth analyzing these requirements in the context of residential buildings and comparing the outcomes with the existing ventilation rates of the analyzed residential building. The floorplan of the first floor consists of 15 zones as shown in Figure 4. Assuming that we have one patient in each zone, that means the total required ventilation rate for the building in this case should be around 2400 L/s which is simply 15 patients times 160 L/s/patient for the worst-case scenario. Converting the volume flow rate (2400 L/s) to air change rate give us 8 ACH which is achievable by natural ventilation in case if the windows are kept open at 20% opening fraction or greater as shown in Figure 6.

In general, the above discussion could be an evidence of the effectiveness of natural ventilation and the subsequent high ventilation rates to provide high dilution capability and minimizing airborne cross-infection transmission. However, further analysis is required to confirm the correlation between air change rates and cross-infection risk in the historical residential buildings in Jeddah. Consequently, the Wells-Riley equation [62], which is a classical mathematical model used for predicting the probability of infection and the risk of airborne disease transmission, has been used. The Wells-Riley equation is presented as follows:

(1) $$P=1-e^{-Iqpt/Vn}$$

where *P* is the probability of infection transmission, *I* is the number of patients (infection source), *q* (quanta/h) is the quantum generation rate produced by an infector, *p* (m^3^/h) is the respiratory ventilation rate of each susceptible, *t* (hours) is the exposure time, *V* (m^3^) is the volume of the enclosed space, and *n* (ACH) is the air change rate of the ventilation system.

Initially, the number of source patients (*I*) is assumed to be 1. A recent research study by Dai and Zhao [63] introduced the range of quantum generation rate for COVID-19 through regression analysis using known quantum generation rates and basic reproductive numbers of other airborne transmitted infectious diseases from previous studies. The range of quantum generation rate (q) for COVID-19 according to Dai and Zhao [63] is found to be between 14 and 48 (quanta/h). The respiratory ventilation rate (*p*) is assumed to be 0.3 m^3^/h which corresponds to occupants with indoor light activity. For this analysis, the duration of exposure (*t*) is estimated as 24 h. The volume of the first floor of the building, which is the analyzed area, is 1080 m^3^. Table 1 summarizes the values of the parameters used in the analysis to predict the probability of infection.

**Figure 7:**
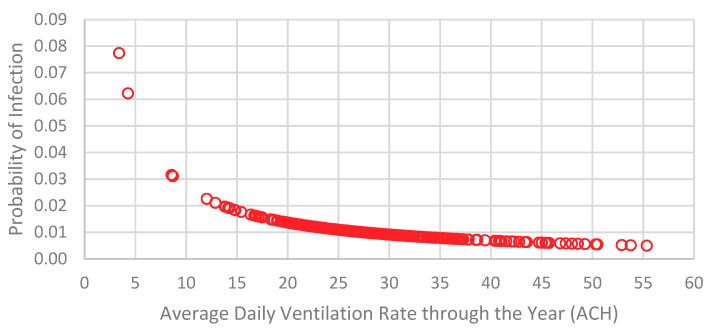
Impact of ventilation rates on the probability of infection for the selected historical house in Jeddah.

The average daily ventilation rate values of the whole year predicted for the fully opened windows case has been substituted in the Wells-Riley equation, and the impact of the ventilation rates on the probability of infection inside the building is computed. The results show an exponential decay where the probability of infection is diminished with the increase of ventilation rate as illustrated in Figure 7. Significant low probability of infection is observed for low ventilation rates. For instance, a ventilation rate of 3 ACH will lead to 0.07 probability of infection which is still considered insignificant.

**Figure 8:**
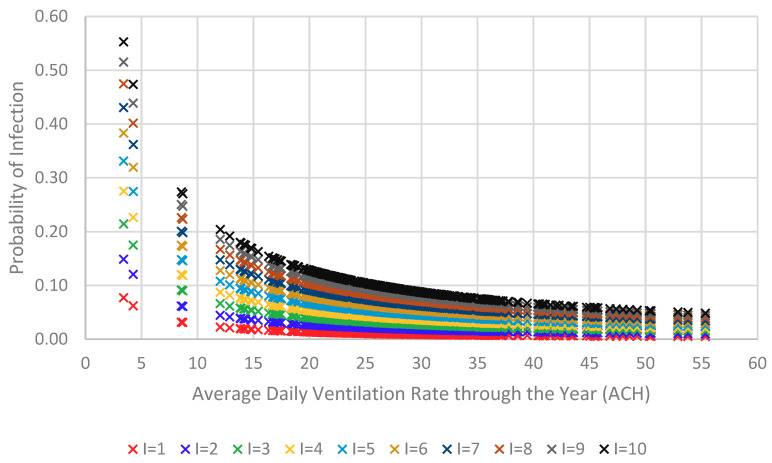
Impact of number of patients on the probability of infection for the naturally ventilated historical house in Jeddah.

The results shown in Figure 7 is when considering only one source patient, thus, sensitivity analysis is performed to investigate the effect of the number of source patients on the probability of infection. As the results in Figure 8 suggest, the higher the ventilation rate the lower the probability of infection. Indeed, for this particular historical house, airborne infection can be minimized significantly when providing air change rates of 25 ACH and higher even with high numbers of source patients as illustrated in Figure 8.

**Figure 9:**
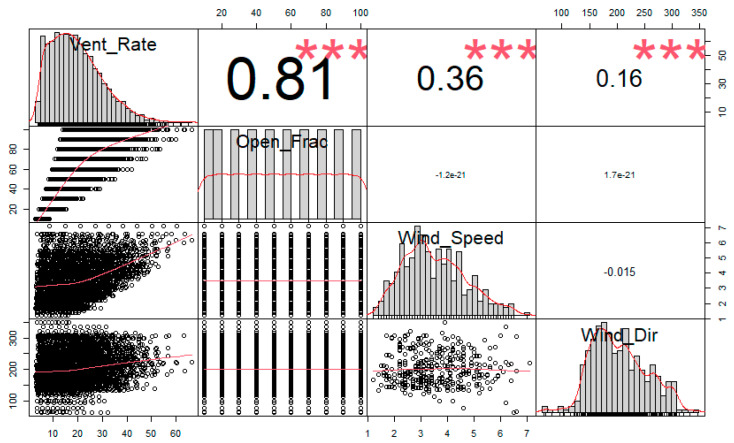
Scatter plot and correlation of the indoors ventilation rate against window opening fraction, wind speed and wind direction. Note: *** Vent_Rate: Ventilation Rate, Open_Frac: Opening Fraction, Wind_Dir: Wind Direction.

A series of simulation runs have been conducted using EnergyPlus to predict the average air change rate inside the building for various values of the following variables: opening fraction of the windows, wind speed and wind direction. The values of the window opening fraction ranges between 10% to 100%, while the values of wind speed and wind direction corresponds to all the available hourly values in the TMY weather file for Jeddah. Figure 9 shows scatterplots and correlations for the variation of ventilation rates as a function of window opening fraction, wind speed and wind direction. The regression analysis presented in Figure 9 clearly indicate a relatively high correlation between the ventilation rate and window opening fraction with 0.81 correlation value as stated in Figure 9. On the other hand, low correlation values were observed for wind speed and wind direction where the estimated correlation values are found to be 0.36 and 0.16 for the wind speed and wind direction respectively. The size of some of the zones and the size of the windows could be the reason behind these low correlations between ventilation rates and both wind speed and wind direction. As discussed previously, most of the wind in Jeddah comes from the north west resulting in a high air change rate in the zones located on that side of the building; however, small zones with large windows on the east and south sides of the building could have very high air change rates which weakens the correlation between ventilation rate and wind direction. Nevertheless, this could be an encouraging sign indicating high air change rates in all building zones due to floorplan design, zone sizes and window to wall ratio, in addition to large openings between the interior zones.

A stepwise regression analysis is carried out to develop a fit model that can predict the average air change rate of the historical building in Jeddah using three independent variables: opening fraction of the windows, wind speed and wind direction. Equation (2) provides the regressed correlation for the air change rate of the building:

ACH = β_0_ + β_1_OF + β_2_WS + β_3_WD + β_4_WS.WD + β_5_OF^2^ + β_6_WD^2^ (2)

where OF is the opening fraction of the window; WS is the wind speed; and WD is the wind direction. The regression model coefficients are listed in Table 2. The correlation regression R^2^ equals to 0.82. A prediction verification analysis of the regression model outlined in Equation (2) has been performed. The verification results indicate that the regression model is able to provide acceptable predictions when compared to the simulation results as expressed by the value of the root mean square error (RMSE) which equals to 6.7.

### CFD Analysis

The simulation results presented above showed an evidence of the effectiveness of natural ventilation on reducing the risk of airborne infection transmission for the selected historical residential building in Jeddah. However, to investigate the details of airflow distribution and the age of air inside the building, CFD simulation has been conducted using the CFD package in DesignBuilder [64]. The DesignBuilder’s CFD package has been validated against experimental measurements in a natural cross-ventilation setting by Baharvand et al. [65]. The validation study showed good agreement for indoor air temperatures and air velocity, thus, the DesignBuilder’s CFD package claimed to be capable of producing accurate predictions for natural ventilation analysis. Moreover, DesignBuilder’s CFD has been verified against Phoenics, a widely respected commercially available CFD package [66]. The verification study showed that DesignBuilder is able to predict the same simulation results as Phoenics when applying the same input data [67].

**Figure 10:**
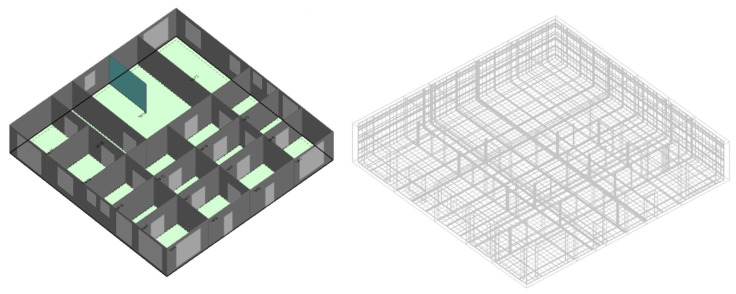
Computational domain and the surface grid of the CFD model.

For this particular analysis, DesignBuilder [68] was selected in order to export the temperature and airflow rates boundary conditions produced by EnergyPlus simulations and share it with the CFD module. It is worth mentioning that EnergyPlus is the energy simulation engine utilized by DesignBuilder to perform hourly simulations of energy models. For CFD boundary conditions, all surfaces and sub-surfaces temperatures in addition to the airflow in and out of each external and internal windows have been exported as a CFD boundary conditions to represent the exact environmental conditions of a specific hour for a selected day (1 March at 17:00). CFD boundary conditions of selected building surfaces for each orientation are summarized in Table 3. Note that mass flow rate balance should be fulfilled for each zone. Hence, a zero flowrate (in or out) in some instances, as presented in Table 3, means that there is another window in the zone that should balance the mass flow rate inside the zone. The standard k-ε model has been selected to account for the turbulence effect due to its ability to offer reasonable accuracy at low computational cost especially when analyzing natural ventilation for the indoors [69,70,71]. The SIMPLER algorithm is adopted to solve the coupled mass and momentum equations in addition to the pressure equation. The first order upwind differencing scheme has been used as the discretization method. The number of iterations has been set to 10,000 due to the complexity of the computational domain, while the convergence criterion has been set to 0.001%. Grid independency is applied to guarantee that the size of the mesh will not affect the accuracy of the results. Based on the grid independency results, the mesh with the 49,060 cells is sufficient for reliable predictions and acceptable computational cost thus selected as the final grid size for the CFD simulation. Figure 10 illustrate the computational domain and the surface grid of the CFD model.

**Figure 11:**
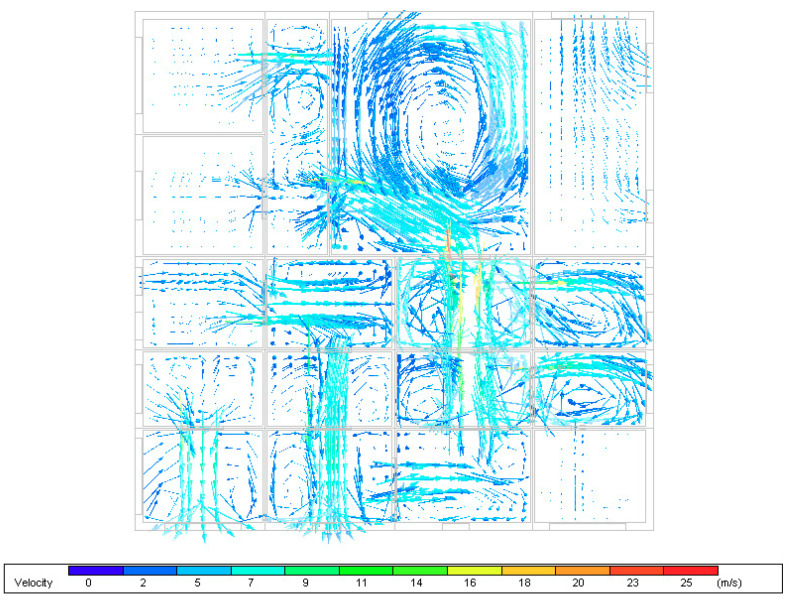
Velocity and airflow patterns inside the building zones.

The simulation was conducted for 1 March at 5 pm where wind speed is at its peak. Figure 11 shows the velocity vectors inside the building zones. The airflow patterns in the zones indicate an adequate air distribution through the areas of all spaces except for a few zones especially the one located on the south east side which could be the cause of wind availability. It is clear from Figure 11 that the interior openings between the spaces are helping in evenly distributing airflows around the spaces with acceptable air velocity that ranges between 1 m/s up to 15 m/s in some areas. The average air velocity inside the building is around 4.5 m/s.

**Figure 12:**
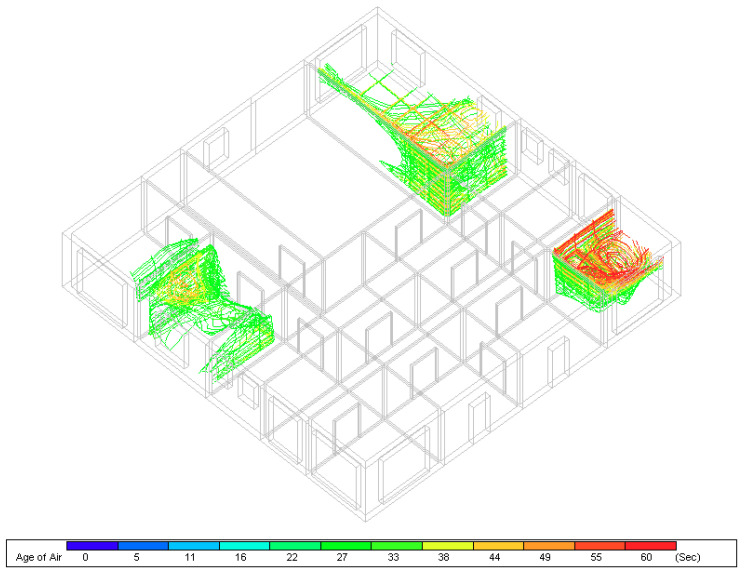
Age of air inside the building zones.

Age of air inside the building spaces has been computed to monitor the duration of air presence inside the spaces. Indeed, the shorter the time of air presence in the indoors the higher the availability of fresh air which means faster dilution of contaminated air. As illustrated in Figure 12, air that is replaced in less than twenty seconds is removed while air with longer durations is shown to spot areas with still air if any. According to the simulation results shown in Figure 12, the majority of the building spaces replace air in less than twenty seconds. On the other hand, a few zones have been spotted that contain air flowing in the same space for longer than twenty seconds. Nevertheless, the longest time for air to stay inside the building does not exceed one minute which is still counted as low. Based on the above discussion, it has been clear that natural ventilation is working effectively by producing acceptable ventilation rates and providing adequate airflow distribution in the selected building that will certainly contribute to enhancing indoor air quality while minimizing airborne infection transmission.

## 4. Concluding Remarks

The strategies to control and prevent the spreading of airborne diseases in a closed environment involve an adequate assessment of threats and resources, then applying the appropriate controls. Buildings typically recirculate some air, which has been shown to promote higher risks of infection spread, as contaminated air in one area is circulated to other parts of the building. Therefore, the required controls include environmental and other engineering controls in conjunction with using a suitable ventilation system. Paying attention to ventilation requirements in enclosed spaces may lead to significant infection-control benefits. There are extensive research work found in the literature that have explored and assessed the effectiveness of various ventilation systems on eliminating the potential of airborne infection transmission in confined spaces; however, there is a lack of research on quantifying the probability of infection as a function of ventilation rate in multi-zone buildings. Thus, the main contribution of this research study, is to present an analysis approach that is able to analyze the impact of ventilation rates on the probability of airborne infection using an existing classical mathematical model developed to predict the probability of infection in buildings as a function of ventilation rates, number of source patients, duration of exposure and other parameters. A historical building (i.e., Hazzazi House) located in Jeddah, KSA has been selected for this analysis. The analysis shows that the large window and wide openings between interior spaces in this historical building assist in providing exceptional cross ventilation conditions which help in producing high indoor air ventilation rates throughout the year. In addition, indoor ventilation rates of 3 ACH is found to be capable of minimizing the probability of infection to 0.07. For this particular historical house, airborne infection can reach to insignificant values even with high number of source patients when delivering ventilation rates higher than 25 ACH. On the other hand, extensive simulation runs have been conducted to predict the average ventilation rates as a function of opening fraction of the windows, wind speed and wind direction. Correlation analysis were implemented using the simulation results which resulted in high correlation between ventilation rates and window opening fraction while relatively low correlations were observed for wind speed and wind direction. Nevertheless, the overall high air change rates in all the zones of the Hazzazi House demonstrate the influence of the floorplan design, zone sizes, window sizes and the large openings between interior zones on improving ventilation rates despite the conditions of wind speed and wind directions. The CFD analysis also proved that openings between interior spaces helped in distributing the airflow evenly throughout the building zones with an acceptable average indoor air velocity of around 4.5 m/s. As stated earlier, the analysis approach presented in this paper revealed the significance of natural ventilation in improving indoor air quality and minimizing airborne infection risk; however, one of the limitations of this approach is neglecting the influence of infector’s aerosols circulation in indoor environmental settings. Therefore, Future research could be done on developing a mathematical model that incorporates the impact of aerosols movements on the probability of infection in addition to the other variables presented in this research work.

Further research can be conducted on a variety of other historical buildings located in different regions of Saudi Arabia including Asir Region, Madinah Region, Qassim and Riyadh Region. This allows for studies to be done on areas with different climate conditions and different historical building designs. The diversity of case studies provides a wider range of outcomes that will better support the suggested method that we have adopted in this study. It is to be noted that the government of Saudi Arabia has been recently interested in examining the possibilities of using heritage buildings as quarantine centers, as the demand of quarantine spaces are increasing drastically since the start of the pandemic. Furthermore, studies of these types are still missing in the typical geographic settings in Saudi Arabia. As a result, this study expands opportunities for decision-makers to assess the possibilities of old buildings as healthy location for expanding quarantine centers when required. Consequently, the method adopted in this study is critical to assess the potential exposures of viruses inside the old buildings. Additionally, we are suggesting that spatial variability may influence the model outcome significantly. In this regard, it is highly recommended that the data be collected in a pilot basis before applying the similar model in another geographical setting.

## Figures and Tables

**Table 1:** List of the parameter’s values used in the Wells-Riley equation.

Parameter	Symbol	Value
Number of source patients	I	1
Quanta generation rate	q	48 quanta/h
Pulmonary ventilation rate	p	0.3 m^3^/h
Duration of exposure	t	24 h
Space volume	V	1080 m^3^

**Table 2:** The values of the regression model coefficients.

β_0_	−21.81
β_1_	0.344
β_2_	1.53
β_3_	0.117
β_4_	0.0086
β_5_	−0.0004
β_6_	−0.00027

**Table 3:** Summary of the CFD boundary conditions of selected surfaces for each orientation of the building model.

Building Surface	Orientation	Boundary Condition
Wall	North	25.7 °C
Window		Flow in = 1571 L/s Flow out = 0 L/s
Wall	South	29.6 °C
Window		Flow in = 75 L/s Flow out = 598 L/s
Wall	West	28.2 °C
Window		Flow in = 599 L/s Flow out = 186 L/s
Wall	East	28.4 °C
Window		Flow in = 0 L/s Flow out = 3778 L/s
Average indoor air temperature	-	26.7 °C
Outdoor air temperature	-	26 °C

## Data Availability

The data presented in this study are available upon request from Alaa Alaidroos. The data are not publicly available due to the heritage building protection privacy systems.
